# Inferior Turbinate Hypertrophy: A Comparison of Surgical Techniques

**DOI:** 10.7759/cureus.32579

**Published:** 2022-12-15

**Authors:** Ioannis Karamatzanis, Panagiota Kosmidou, Vasiliki Ntarladima, Beatrice Catalli, Anastasia Kosmidou, Dimitrios Filippou, Christos Georgalas

**Affiliations:** 1 Internal Medicine, University of Nicosia Medical School, Nicosia, CYP; 2 Otolaryngology - Head and Neck Surgery, Mediterranean Hospital of Cyprus, Limassol, CYP; 3 Otolaryngology - Head and Neck Surgery, University of Patras, Medical School, Patras, GRC; 4 Otolaryngology - Head and Neck Surgery, Evangelismos General Hospital, Athens, GRC; 5 Internal Medicine, Università Vita Salute San Raffaele, Milan, ITA; 6 Pulmonology, Sotiria General Hospital of Thoracic Diseases, Athens, GRC; 7 Surgery, National and Kapodistrian University of Athens Medical School, Athens, GRC; 8 Otolaryngology - Head and Neck Surgery, Hygeia Hospital, Athens, GRC; 9 Surgery - Head and Neck, Nicosia University, Nicosia, CYP

**Keywords:** nonallergic rhinitis, allergic rhinitis (ar), unilateral nasal obstruction, nasal obstruction surgery, nasal obstruction, inferior turbinate, surgical endoscopy

## Abstract

Introduction

Nasal obstruction is one of the most frequently reported symptoms in clinical practice. The second most common cause of nasal obstruction is inferior turbinate hypertrophy, a nasal pathology for which surgical treatment is often required. This study aims to determine the most effective surgical method in patients with inferior turbinate hypertrophy (ITH).

Materials and methods

The study was performed from September 2018 to October 2019 in the Otolaryngology-Head and Neck Surgery Department of the Evangelismos Hospital of Athens. The study population comprised 205 patients that underwent surgery and were monitored in the hospital. Radiofrequency ablation (RFA) was the method used in 73 patients, 68 patients were treated with the microdebrider-assisted turbinoplasty (MAT), and the remaining 64 patients were operated on using electrocautery (EC). Following surgery, postoperative complications were assessed and quantified.

Results

Overall, 205 patients underwent surgery. The first group (n=73) was operated on using radiofrequency ablation and had a complication rate of 30.1%. Out of 73 patients, 51 recovered without complications. The remaining 22 had complications, consisting of 16 patients with bleeding and six with postnasal drip.

The second group (n=68) was treated using the microdebrider method. The complication rate was 26.5%, where 50 patients did not present with any symptoms post-operatively and 18 exhibited symptoms. Specifically, postnasal drip was more prevalent with this method as all 18 patients showed postnasal drip as their complication.

The third group (n=64) was treated with electrocautery. Patients in this group had the most complications (n=24), 16 were attributed to postnasal drip and eight to infections, treated promptly with oral antibiotics. The complication rate using this method was 37.5%.

Conclusion

In our study, the microdebrider-assisted turbinoplasty offered the lowest complication rate, followed by radiofrequency ablation and electrocautery. However, all three methods managed to alleviate the nasal obstruction and treat inferior turbinate hypertrophy. More research is needed as a lack of consensus remains regarding the optimal surgical technique for lower turbinate hypertrophy.

## Introduction

Nasal obstruction is one of the most frequently reported symptoms in clinical practice, with up to one-third of the population affected [1]. It may present as bilateral or unilateral, acute or chronic, continuous or transient [2]. The obstruction can cause discomfort, which manifests as a sensation of insufficient airflow through the nose [1]. Chronic difficulty in breathing has a deleterious effect on the quality of life while patients complain of inability to sleep well at night, somnolence, and poor concentration [3]. The most common cause of nasal obstruction is nasal septum deviation, followed by inferior turbinate hypertrophy (ITH) [4]. Inferior turbinate hypertrophy may arise as a response to a septal deformity, known as compensatory hypertrophy [5]. Other causes of ITH include allergic rhinitis, vasomotor rhinitis, and chronic hypertrophic rhinitis [6].

The nasal turbinates are important anatomical structures that arise from the cartilaginous nasal capsule. The inferior nasal turbinate is a separate bone formed from the maxilloturbinal structure at the 10th week of gestation [7]. The function of the nasal turbinates is to warm and humidify air entering the nasal cavity, and their curvature increases the overall surface area to assist in this function [8]. The inferior turbinates alter their size by contracting or enlarging to permit or obstruct airflow, which regulates the moisture levels [8].

Inferior turbinate hypertrophy is defined as the enlargement of the turbinate that may involve the bone and the mucosa [3]. The main etiologies of ITH include septal deviation, allergic rhinitis, vasomotor rhinitis, and chronic hypertrophic rhinitis. Septal deviations are unilateral, most commonly when caused by injury [9,10]. Allergic and nonallergic rhinitis manifest as bilateral turbinate enlargement, which is caused by tissue oedema, cellular hyperplasia, and vascular congestion [11]. Allergic rhinitis (AR) is an atopic disease characterized by sneezing and rhinorrhea, and when chronic, nasal congestion and obstruction are observed [12]. Turbinate hypertrophy in chronic AR is hypothesized to occur due to two main reasons: dilated submucosal venous sinuses becoming varicose and unresponsiveness to sympathetic nervous system stimulation [10].

Furthermore, vasomotor rhinitis is the most common form of nonallergic rhinitis, which is defined as inflammation of the nasal mucosa without clinical evidence of endonasal infection or systemic signs of sensitization to inhalant allergens [13]. The causative mechanism is neurogenically mediated, with dysregulation of sympathetic and parasympathetic nerves, responsible for the vascular tone and mucus secretion, respectively [13]. As in AR, the increased vascular permeability and mucus secretion lead to congestion and nasal obstruction. Finally, chronic hypertrophic rhinitis is defined as hypertrophy, thickening and swelling of the mucous membrane, with at least two nasal symptoms, including rhinorrhea, blockage, sneezing or itching for more than two weeks [14].

Initial management for nasal obstruction is conservative. The first-line therapy is antihistamines, systemic or topical decongestants and corticosteroids [15]. However, symptoms persist for many patients, and the next step is surgery. The treatment for ITH is tissue-volume reduction surgery, a procedure aimed at reducing the volume of the mucosa of the lower nasal turbinates [15]. Depending on the method, the surgery targets both the bone and submucosal tissue. The fibrosis on the submucosal tissue minimizes engorgement of the turbinates, while the bone reductions increase the intranasal space, relieving the nasal obstruction [16].

The high prevalence of the disease results in many surgeries being performed, which led to the introduction of novel, non-invasive methods to ensure good functional outcomes with lower rates of complications. For this reason, in recent years, radiofrequency ablation (RFA), microdebrider-assisted turbinoplasty (MAT), and to a lesser extent, electrocautery (EC) have been the primary methods utilized. They have proven effective in dealing with functional problems, minimizing hospitalization time, and reducing complications [17].

Radiofrequency ablation uses heating to create ionic change, which increases local temperature and generates profound thermal tissue destruction [4]. The resulting fibrosis reduces tissue volume and alleviates nasal obstruction [18]. Microdebrider-assisted inferior turbinoplasty is a newer, minimally invasive method for reducing inferior turbinate size while maintaining mucosal integrity [19]. Finally, electrocautery, the oldest technique to date, utilizes thermal energy to induce coagulation and thus reduces the vascularity and volume of the tissue [20]. Lower turbinate surgery seeks to conserve the mucosal lining, ensuring normal function, quicker recovery, and less bleeding and preventing atrophic rhinitis [21].

This study aims to determine the most effective surgical method in patients with inferior turbinate hypertrophy.

## Materials and methods

The Institutional Review Board (IRB) of the National and Kapodistrian University of Athens, Greece, issued approval number 9126 for this retrospective study. 

Data collection

The study was conducted from September 2018 to October 2019 in the Otolaryngology-Head and Neck Surgery Department of the Evangelismos Hospital of Athens. Each patient obtained a consent form, and data confidentiality and protection were maintained. The inclusion criteria for our study included patients with inferior turbinate diseases that required surgical management.

This study comprised 205 patients that underwent surgery and were monitored in the hospital. Radiofrequency ablation was the surgical modality used in 73 cases, 68 patients were treated with the microdebrider approach, and the remaining 64 patients were operated on using electrocautery. The postoperative complications, according to each method, were assessed and quantified. The follow-up of patients was done two weeks after the surgery.

Statistical analysis

Data were collected using Excel software (Microsoft Excel, Microsoft® Corp., Redmond, WA) and analyzed using SPSS v20 software (IBM Corp., Armonk, NY). To determine the relationship between the percentage of complications related to the different surgical modalities, the Chi-square test was carried out. A value of p < 0.05 was accepted as statistically significant.

## Results

Overall, 205 patients underwent surgery for inferior turbinate hypertrophy refractory to medical therapy (Table 1). The first group (n=73) was operated using radiofrequency ablation. Out of 73 patients, 51 recovered and had no complications. The remaining 22 presented with complications, of which 16 experienced bleeding, and six reported postnasal drip (Table 2).

**Table 1:** The number of patients operated on with each surgical modality.

Method					
		Frequency	Percent	Valid Percent	Cumulative Percent
Valid	Radiofrequency ablation	73	35.6	35.6	35.6
	Microdebrider	68	33.2	33.2	68.8
	Electrocautery	64	31.2	31.2	100.0
	Total	205	100.0	100.0	

**Table 2:** Complication type related to the surgical modality

		Method			Total
		Radiofrequency ablation	Microdebrider	Electrocautery	
Complication type		51	50	40	141
	Postnasal drip	6	18	16	40
	Bleeding	16	0	0	16
	Infection	0	0	8	8
Total		73	68	64	205

**Figure 1:**
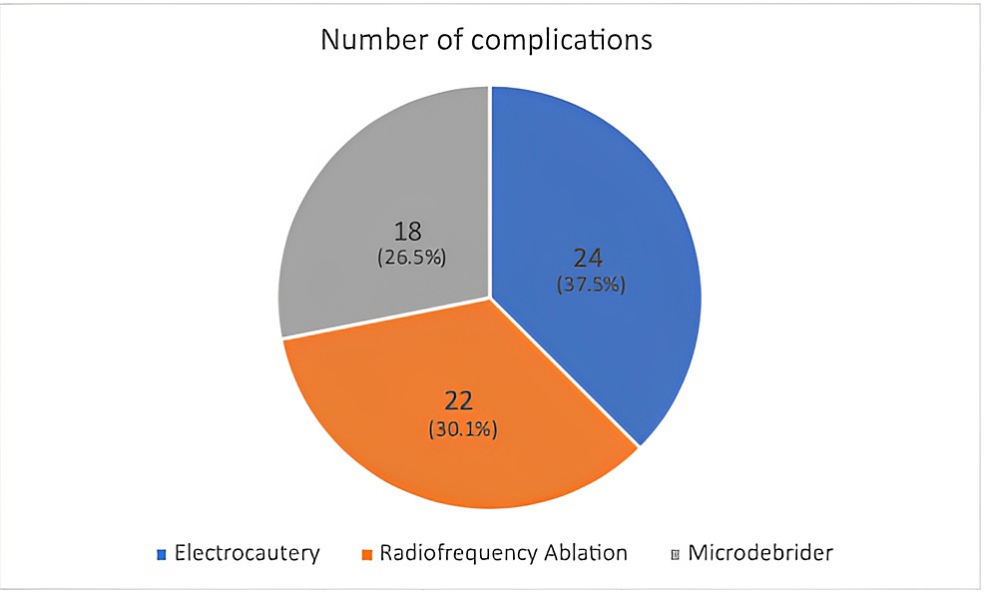
Surgical modality used and the number of complications it produced

The second group (n=68) was treated surgically using microdebrider-assisted turbinoplasty (MAT). The complication rate of MAT was 26.5%. More specifically, 50 patients reported no adverse event, while 18 exhibited postnasal drip as a complication. Among the three treatment groups, the patients treated with MAT had the highest reported number of postnasal drips as an adverse event but the lowest overall complication rate (Figure 1).

Furthermore, the Chi-square test was performed to assess the relationship between the complication rates and the different surgical modalities used (Table 3). The p-value was 0, less than 0.05; thus the results are statistically significant.

**Table 3:** Chi-Square Test a: Four cells (33.3%) have an expected count of less than 5. The minimum expected count is 2.50. df: Degrees of freedom

	Value	df	p-value
Pearson Chi-Square	57.235^a^	6	.000
Likelihood Ratio	63.870	6	.000
N of Valid Cases	205		

The last cohort, which was treated with electrocautery (n=64), presented with the highest number of complications (n=24) and the highest complication rate of 37.5% (Table 4 ). Of the 24 complications, 16 were attributed to postnasal drip and eight to infections, treated promptly with oral antibiotics. The complication rate using this method was 30.1% (Table 4). 

**Table 4:** Complication rate of each surgical modality.

	Frequency	Without complications	With Complications	Complication Rate(%)
Radiofrequency ablation	73	51	22	30.1
Microdebrider	68	50	18	26.5
Electrocautery	64	40	24	37.5
Total	205	141	64	-

## Discussion

Nasal obstruction secondary to nasal turbinate hypertrophy is a prevalent condition that impacts the patient’s quality of life. The causes of ITH vary; the most common include septum deviation (compensatory hypertrophy), allergic rhinitis, vasomotor rhinitis, and chronic hypertrophic rhinitis. The first-line therapy is antihistamines, systemic or topical decongestants and corticosteroids [15]. In addition, several surgical techniques are available to address ITH in patients refractory to medical therapy. These include conventional turbinoplasty, microdebrider submucosal resection, laser turbinectomy, radiofrequency turbinoplasty, and electrocautery turbinectomy [22]. Older surgeries, such as aggressive turbinectomy, may give better long-term results but have a higher risk of complications such as crusting and epistaxis [17]. New techniques allow for better outcomes, fewer postoperative complications, and faster recovery [22]. However, a lack of consensus remains regarding the optimal surgical technique to treat lower turbinate hypertrophy.

Surgeries for nasal turbinate reduction have been categorized as mucosal and non-mucosal preservation surgery. The techniques aim to balance turbinate size reduction and nasal obstruction relief with the preservation of mucosal tissue function to ensure long-term results and reduce postoperative complications [22]. Mucosal preservation surgery includes conventional turbinoplasty, microdebrider turbinoplasty and radiofrequency turbinoplasty [22]. Non-mucosal preservation techniques comprise laser turbinectomy, conventional turbinoplasty, electrocautery turbinectomy and cryoturbinectomy [22]. The latter category of surgeries represents a less-mucosal-friendly approach, which results in significant tissue loss and various postoperative complications such as crusting, excessive bleeding and longer recovery. Therefore, postoperatively, mucosal-preserving surgery is preferred to reduce complications and maintain tissue patency. 

Electrocautery (EC), the oldest method used, induces a localized heating effect that causes a reduction in the vascularity and volume of tissue [23]. Subsequently, submucosal thermal lesion leads to fibrosis, scar contraction, and tissue volume reduction [17]. In electrocautery, both bipolar and monopolar devices are used. Still, the former is preferred as the burn's depth and thermal injury is less, and the accidental heat dispersal is reduced [17]. A systematic review of 1231 patients that underwent surgery with EC achieved an overall decrease in nasal resistance, and partial or complete resolution of nasal obstruction was seen in 67.3% of participants [17]. The most common postoperative complication reported was nasal bleeding and crusting [17]. In our study, 64 patients received surgery using electrocautery, where post-operative recovery was unremarkable in 40 (62.5%). However, 24 patients (37.5%) exhibited complications, the highest number in our study. In detail, 16 patients had postnasal drip shortly after surgery and eight had infections that resolved with oral antibiotics. Despite the complications, rapid healing and early restoration of tissue function were observed. Finally, Uluyol et al. [20] found that EC has a lower economic burden than RF.

Radiofrequency ablation (RFA) or radiofrequency tissue reduction was introduced in the early 2000s and is a commonplace technique used to treat ITH. The radio frequencies emitted create ionic change, resulting in a focal well-controlled temperature increase, generating deep thermal tissue destruction [4]. Similarly to EC, the resultant tissue fibrosis and scarring by RF decrease the turbinate size and alleviate nasal obstruction [18]. RF has many advantages, including preservation of nasal mucosa, a low rate of complications, and a significant reduction in the severity and frequency of nasal obstruction [24]. A recent systematic review by Sinno et al. [17] evaluated the use of RF in 21 studies comprising 1515 patients, and resolution or significant improvement in nasal airflow was found in 85.5% of patients. The most commonly reported complications were bleeding and postnasal drip [17]. In one study, long-term follow-up of patients treated with radiofrequency tissue reduction showed partial or complete relief of symptoms in 85% of participants up to 30 months [25]. However, most studies offer preliminary results, short follow-up and are observational [24]. Moreover, for patients with prolonged mucosal hypertrophy, the tissue fibrosis induced by the radiofrequency ablation technique may be insufficient to cause a reduction of the turbinate structure [26]. In our study, in the group treated with RF, a total of 22 patients exhibited complications. Postoperative bleeding was observed in 16 patients, and six had a postnasal drip, with a complication rate of 30.1%.

The last method we assessed in our study was microdebrider-assisted turbinoplasty (MAT), a recent, minimally invasive endoscopic procedure for reducing inferior turbinate size while maintaining mucosal integrity [19]. This technique utilizes a micro excision device (shaver) with a rotating blade that shears the tissue. The microdebrider also contains a real-time suction, which traps the tissue downstream, allowing for histopathological analysis [27]. MAT has gained traction recently as it has many benefits, including a short healing time, minor postoperative problems, and an excellent functional outcome [28]. A meta-analysis by Mirza et al. [29] found that MAT is a better-outcome surgical procedure than RF in managing nasal obstruction caused by enlarged inferior turbinates. Lorenz and Maier [28] found that microdebrider turbinoplasty is associated with minor trauma to the mucous membranes, and patients reported markedly lower average complaints and pain scores [28]. A study comparing all current surgical methods to treat inferior turbinate hypertrophy found that overall microdebrider-assisted resection had the lowest complication rates [17]. Our study concurs with the above, as the patients treated with the microdebrider surgery had the lowest complication rate of 26.5%, and all 18 patients experienced postnasal drip.

In our study, the complications rate of the three methods was statistically significant, comparable, and in agreement with the results reported in the medical literature. It can therefore be concluded that MAT had the lowest complication rate, followed by radiofrequency and electrocautery. However, all three methods treated the nasal obstruction, and the postoperative complications resolved within three weeks. In the end, the choice of modality depends on the surgeon's preference and experience in collaboration with the patient's informed decision.

Study limitations

The study has some limitations. Firstly, the small sample size limits our research, and a larger cohort is required to generalize our results. Also, the turbinates' original size and morphology can affect each surgical technique's outcomes, although this is challenging to identify and quantify. Finally, the modality used in each surgery is operator-dependent and relies on the surgeon’s experience and familiarity, which may affect postoperative complications and functional results.

## Conclusions

Surgical treatment of inferior turbinate hypertrophy aims to balance the conservation of mucosal tissue, low complication rates, and preserved turbinate function. In our study, the surgical modality for treating inferior turbinate hypertrophy with the lowest complication rate was microdebrider-assisted turbinoplasty, followed by radiofrequency ablation and electrocautery. All three methods have proven effective in relieving nasal obstruction resulting from turbinate enlargement. However, more research is needed as a lack of consensus remains regarding the optimal surgical technique. Ultimately, the choice of modality depends on the surgeon’s preference and experience in collaboration with the patient’s informed decision.
